# Utilizing Regulatory T Cells Against Rheumatoid Arthritis

**DOI:** 10.3389/fonc.2014.00209

**Published:** 2014-08-08

**Authors:** Mohammad Haque, Kristin Fino, Fengyang Lei, Xiaofang Xiong, Jianxun Song

**Affiliations:** ^1^Department of Microbiology and Immunology, Pennsylvania State University College of Medicine, Hershey, PA, USA

**Keywords:** regulatory T cells, autoimmunity, rheumatoid arthritis, cell-based therapies, suppressive cytokines, stem cells

## Abstract

Regulatory T (Treg) cells are essential for normal immune surveillance systems, and their dysfunction leads to development of diseases, such as autoimmune disorders. CD4^+^CD25^+^ Treg cells are well-known suppressive cells, which express the transcription factor Foxp3, are indispensable for the maintenance of immune self-tolerance and homeostasis by suppressing aberrant or excessive immune response. Other Foxp3^−^ Treg cells include Tr1, Th3, CD8^+^CD28^−/−^, and Qa1-restricted T cells; however, the contribution of these Treg cells to self-tolerance, immune homeostasis as well as preventing autoimmunity is not well defined. Here, we discuss the phenotypes and function of Foxp3^+^ Treg cells and the potential use of such Treg cells against rheumatoid arthritis (RA). Of note, even though most expanded populations of Foxp3^+^ Treg cells exhibit suppressive activity, tissue-associated or antigen-specific Treg cells appear superior in suppressing local autoimmune disorders such as RA. In addition, utilizing tissue-associated Foxp3^+^ Treg cells from stem cells may stable Foxp3 expression and avoid induction of a potentially detrimental systemic immunosuppression.

## Introduction

Rheumatoid arthritis (RA) is a chronic autoimmune disorder, which has approximately 1% prevalence in the industrialized world, and is described by pain and stiffness of the joints, and inflammation of the synovial membrane. This systematic autoimmune disorder results in the accumulation of inflammatory cells such as T cells, B cells, and macrophages in the inflamed joints, which can lead to persistent synovitis and tissue destruction, typically articular cartilage destruction. Being a disabling and painful condition, RA often causes loss of mobility and function, and is commonly accompanied by substantial comorbid conditions in the cardiovascular, neurologic, and metabolic systems (1). The autoantigens are still unidentified in spite that RA is considered as autoimmune disease. The main cause of autoimmune disorder like RA is the failure in the maintenance of immunological self-tolerance. There are multiple mechanisms for maintaining the self-tolerance within the immune system. It is considered that autoreactive T cells and B cells are vital for the pathogenesis of RA. Autoreactive T cells are mainly deleted in the thymus; however, this process is not strict. Nevertheless, autoreactive T cells can escape into the peripheral immune system, and subsequent activation will cause autoimmune pathological disorders (2).

The etiological factor for the development of autoimmune disorder remains unclear; however, the progression of the RA disease is associated with synovial inflammation, which can develop to a Pannus (thickening synovial tissue) and damage the surrounding cartilage and bone. Plasmatic cells infiltrate into the joint and produce antibodies that form aggregates of IgG. In order, the immune system recognizes these IgG aggregates as foreign antigens. Within the synovial membrane, plasmatic cells, T cells, B cells, dendritic cells (DCs), and macrophages develop lymphoid follicle-like constructions, and start to create antibodies against these constructions recognized as rheumatoid factor RF (3). The IgM class is the most significant type of RF in regards to RA that emerges in 70–80% of patients. During the development of the disease, a wide variety of cells, including B cells, macrophages, DCs, neutrophils, fibroblasts, and granulocytes profoundly infiltrate into the normal and relatively avascular synovium. However, the concept for the pathogenic events that are critical for the triggers for the onset of diseases remains undetermined. In general, RA is induced by interactions of multiple factors, including genetic, biomechanical, and environmental factors, neuro-immune interactions, and impaired articular microvascular function (4). Up to now, by using the genome-wide association studies, a number of genetic loci have been suggested to be associated with RA susceptibility and severity (3, 5, 6).

The immune system can defend against pathogenic attack, and is responsible for maintaining effective immune response as well as promoting an adequate inflammatory response. Regulatory T (Treg) cells, formerly known as suppressor T cells, are a developmentally and functionally distinct T cell subpopulation that modulates the immune system, retains tolerance to self-antigens, and eliminates autoimmunity. CD4^+^CD25^+^ suppressor T cells are well-known Treg cells that express the transcription factor forkhead box P3 (Foxp3), are indispensable for the maintenance of immune self-tolerance and homeostasis by suppressing aberrant or excessive immune response. Other Foxp3^−^ suppressor T cells include Tr1, Th3, CD8^+^CD28^−/−^, and Qa1-restricted T cells; however, the contribution of these Treg cells to self-tolerance, immune homeostasis as well as preventing autoimmunity is not well defined. The main functions of Foxp3^+^ Treg cells are to migrate into inflammation sites and suppress various effector lymphocytes, especially the subsets of CD4^+^ helper T (Th) cells: Th1, Th2, Th17, and follicular Th cells. Over the past decade, a number of studies have addressed that the majority of the Foxp3^+^ Treg cells are produced in the thymus as an antigen-primed and functionally mature T cell subpopulation specialized for immune suppression. However, some of Foxp3^+^ Treg cells differentiate from naive conventional T cells in the periphery under certain condition (7). The characteristics of different Treg cell subsets are briefly described in Table 1.

**Table 1:** **Characteristics of different Treg cell subsets**.

Cell type	Phenotype	Mechanism	Origin
nTreg cells	Foxp3^+^CTLA4^+^TNFR	Contact dependent, CTLA4, IL-10, and TGF-β1	Thymus
	SF18^+^IL7R^low^
	CD25^hi^ (mouse)	
	CD25^very hi^ (human)	
nTreg cells (activated)	Foxp3^+^HLA^−^	Contact dependent	Expansion of natural Treg cells
	DR^+^CD69^+^
	Granzyme B^+^ (mouse)	
	Granzyme A^+^ (human)	
iTreg cells	Foxp3^+^CTLA4^+^TNFR	Contact dependent and in some cases TGF-β1 dependent	Conversion and/or expansion of naive CD4^+^ T cells
	SF18^+^
TH3 cells	Foxp3^−^	TGF-β and or/IL-10	Periphery
Tr1 cells	Foxp3^−^	IL-10	Periphery
	IFNγ^−^(mouse)	
	IFNγ^low^ (human)	
CD8^+^ T cells	CD28^+/−^	Cell contact^−^, LILRB4^−^, and LILRB2 dependent	Periphery

## CD4^+^CD25^+^ Treg Cell Function in Rheumatic Disease

Thymic selection results in the appearance of T cells with two types of T cell receptor (TCR). The majority express αβ chains in the TCR, forming unique structure on each T cell. αβ T cells represent mature T cells that circulate through the secondary lymphoid organs and develop adaptive immune response. Some other fraction of T cells express γδ chains in TCR, reside in skin and mucosal surfaces that play a role in the initial response to microbial invasion. On the basis of lineage marker and functional activities, αβ T cells are subdivided into several groups, including CD4^+^ and CD8^+^, or naive, effector, and memory T cells. Naive T cells are the most homogenous representative of CD4^+^ and CD8^+^ subsets. The activated CD4^+^ T cells can be subdivided into Th1, Th2, Th17, and Treg cell subsets based on production of signature cytokines.

There are two main classes of CD4^+^CD25^+^ Treg cells: naturally occurring Treg (nTreg) cells and induced or adaptive Treg (iTreg) cells. nTreg cells are CD4^+^CD25^+^Foxp3^+^ Treg cells that develop in the thymus. iTreg cells are induced Foxp3^+^ Treg cells converted from Foxp3^−^ T effectors (Teffs) (8). There are multiple signaling pathways that are involved in the activation and control of T cell responses. There are no unique surface markers for Treg cells; however, Treg cells are usually distinguished by the expression of CD25 (the alpha chain of the IL-2 receptor), CTLA-4 (CD152, cytotoxic T-lymphocyte antigen 4), and glucocorticoid-induced tumor-necrosis-factor-receptor-related protein (GITR). These proteins are expressed not only on Treg cells but also on activated T cells that make complications for their use as markers of Treg cells. It remains unclear how the Treg cells are generated and the mechanisms that relate to their function. Nevertheless, CTLA-4 and TGF-β are critical in activity of Treg cells. CTLA-4^−/−^ or TGF-β^−/−^ mice have a more extensive pathology than those lacking CD4^+^CD25^+^ Treg cells, although such Treg cells can function in the absence of TGF-β production or signaling. In general, the signaling mechanisms by which Treg cells are developed and exert their suppressive function still need to be determined.

The transcription factor Foxp3 is highly expressed in lymphoid tissues such as CD4^+^ T cells; however, approximately undetectable in B cells and CD8^+^ T cells. Foxp3 is similar to other genes such as CTLA-4, OX40, and GITR, is a gene associated with cell activation and the Treg lineage. Foxp3 is not expressed in CD4^+^CD25^−^ Th cells upon stimulation. Foxp3 is the most specific gene for the lineage of Treg cells as compared with other associated genes like GITR or CTLA-4. Being an X chromosome-encoded transcription factor, Foxp3 is indispensible for both development and function of Treg cells. Mice mutated in Foxp3 as well as patients with immune dysregulation polyendocrinopathy, enteropathy, and X chromosome-linked syndrome (IPEX) result in the development of complex autoimmune diseases due to the deficiency of Treg cells. When manipulated to ectopically express Foxp3, T cells acquire the phenotype of Treg cells. Furthermore, approximately 90% decrease of Foxp3 protein expression due to destabilizing alterations in the 3′ UTR of the Foxp3 messenger RNA (mRNA), thereby destabilizing mRNA, results in significantly impaired Treg cell-mediated suppression, demonstrating that the amount of Foxp3 protein directly correlates to the function of Treg cells (9). Constitutive expression of Foxp3 is fundamental for the maintenance of the suppressive function of Treg cells (10).

Although the precise signaling mechanisms regulating Foxp3 expression are not fully understood, TGF-β, IL-2, or TCR stimulation of T cells results in increased Foxp3 expression. This is most likely modulated by the demethylation of the Foxp3 promoter or conserved non-coding regions in the Foxp3 locus (11). In addition, multiple transcription factors, including cAMP response element modulator (CREB)/activating transcription factors (ATF), Ets-1 (protein C-ets-1), forkhead box protein O1 (Foxo1), forkhead box protein O3 (Foxo3), and signal transducer and activator of transcription 5 (STAT5), regulate Foxp3 transcription (12, 13). It is not fully clear whether Foxp3^+^ Treg cells can lose Foxp3 expression and suppressive function, as well as whether Foxp3^+^ Treg cells exhibit characteristics of other Th cell subsets. A number of studies in which Foxp3^+^ Treg cells were adoptively transferred into lymphopenic mice demonstrated that approximately 10–50% of the transferred Treg cells lost Foxp3 expression (14–16). Furthermore, Treg cells from both the periphery and the thymus were converted into Th17 cells upon stimulation with anti-CD3, anti-CD28, and IL-6, demonstrating a degree of plasticity (17). In addition, Foxp3^+^ Treg cells can be converted to a Foxp3 Th1 cell phenotype upon *Toxoplasma* infection (18). In fact, Foxp3 can be polyubiquitinated; however, the regulation of this process and the modulators remain elusive (19–21) Deubiquitinating enzyme (DUB) ubiquitin-specific-processing protease 7 (USP7, also known as HAUSP, ubiquitin carboxyl-terminal hydrolase 7 or herpesvirus-associated ubiquitin-specific protease) is active in primary Treg cells and associates with Foxp3. Ectopic expression of USP7 specifically decreased Foxp3 polyubiquitination, resulting in increased Foxp3 expression. Conversely, knockdown of USP7 resulted in decreased Foxp3 expression. Furthermore, the function of Treg cells was noticeably decreased when USP7 was knocked down or when DUB activity was inhibited both *in vitro* and *in vivo* (22). The manipulation of Foxp3 ubiquitination provides a method for temporally controlling the expression of Foxp3 in T cells, thereby regulating the numbers and function of Treg cells.

CD4^+^CD25^+^ Treg cells comprise approximately 5–10% of the mature CD4^+^ T cells in mice and humans, and approximately 1–2% of CD4^+^ Treg cells are detectable in peripheral blood. Murine and human nTreg cells are phenotypically similar on the basis of surface markers, expressing MHC-class II molecules, CD25, CD122, CD132, GITR, CTLA-4, PD-L1, CD62, CD38, CD45RO, and Foxp3. Although both nTreg cells can use the cell–cell contact mechanism to mediate their suppressive function, murine nTreg cells are via a granzyme-B-dependent and perforin-independent pathway, while human nTreg cells are via a granzyme-A and perforin-dependent pathway. Number of CD4^+^CD25^+^ Treg cells in peripheral blood is differed; however, the frequency of Treg cells is constantly higher in the synovial fluid than in the peripheral blood. Furthermore, Treg cells from RA patients still keep their suppressive ability; however, these Treg cells have no capacity to stop the production of inflammatory cytokines such as TNF-α from monocytes or activated T cells (23). This functional defect of Treg cells in RA was related with a high expression of TNF-α that reduced expression of Foxp3, or resulted in defective expression of CTLA-4 (24, 25). Cell number is critical during arthritis development. In many autoimmune disorders, e.g., juvenile idiopathic arthritis, psoriatic arthritis, multiple sclerosis, systemic lupus erythomatosus, autoimmune hepatitis, and type-1 diabetes, the numbers and suppressive activity of circulating CD4^+^CD25^+^ Treg cells dramatically reduced (26). Despite presenting in the joints of patients with RA, Treg cells did not possess normal immune suppressive activity. Treg cells in the synovial fluid from RA patients are exposed to a numbers of inflammatory cytokines; high amounts of TNF-α secreted by the inflamed synovium into the joint fluid likely cause the abnormal phosphorylation of Foxp3, resulting in abnormal suppressive function of Treg cells.

The function and frequency of Treg cells can be measured in peripheral blood as well as at the site of inflammation in arthritic patients. Circulating Treg cells in RA patients holding mutable functional activity, particularly with regard to the suppressive function (27); however, at the inflamed joints, the suppressive activity of enriched Treg cells is high and consistent (28). The general agreement is that these are highly reactive Treg cells, which have an increased suppressive activity (29). Similarly in synovium of RA patients, Foxp3 DNA methylation resulted in a higher dedication toward Treg cell lineage (30). In the inflamed synovium of RA patients, local tissue and different immune cells interrelate through cytokines and/or cell–cell contact. Pro-inflammatory cytokines, e.g., TNF-α, IL-6, and antigen presenting cells (APCs) also affect the function of Treg cells. Despite presenting in large amounts and suppressive function *in vitro*, Treg cells likely display different suppressive capacity *in vivo*, countered by the inflammatory environment, or hampered by the resistance from Teffs (31).

## Modulation of CD4^+^CD25^+^ Regulatory T Cells

A number of therapeutic strategies have been implemented in the clinical and many others are being developed for the treatments of RA including: biological therapy, stem cell therapy, mesenchyme stem cell (MSC)-based therapy, hematopoietic stem cell (HSC)-based therapy, and Treg cell-based therapy. In the field of RA, the biological therapy is a fairly new type of therapeutics, and is usually managed at specific biological targets, which have a function in the inflammatory cascade. In general, these targets are TNF-α, IL-6, the IL-1 receptor antagonist, and soluble CTLA-4. There are three biological agents targeting TNF-α. These are infliximab, adalimumab, and etanercept. Currently, these biological agents are used to treating RA. Despite targeting TNF-α is a successful therapeutic strategy in the treatment of RA, methotrexate (MTX), not targeting TNF-α, is also an effective drug (28). MTX is an antifolate drug that suppresses purine and pyrimidine synthesis and inhibits DNA replication. Early treatment with TNF-α inhibitors combined with MTX has been shown to significantly improve treatments of RA (30, 32). In addition to TNF-α and MTX, there are some other biologic agents for treating moderate to severe RA, including abatacept, rituximab, and tocilizumab (31, 33).

Another effective biological target of RA is IL-6. IL-6 is significantly increased in synovial fluid of RA patients, and functions as a pro-inflammatory cytokine, which directly reduced the suppressive function of Treg cells (23). In addition, IL-6 dramatically influenced the conversion of Foxp3^+^ CD4 T cells into Th17 cells (34). Th17 cells play a crucial role in RA pathology through secreting IL-17. IL-17 has ability to activate a number of cells, such as synovial fibroblasts and monocytes that were involved in causing joint damage (35). Neutralization of IL-17 during reactivation of antigen-induced arthritis in animals prevented joint inflammation and bone erosion (36). Another drug that targets IL-6 receptor is tocilizumab, a humanized antibody that has been proven as a successful therapy for the treatments of RA (37). Tocilizumab can ameliorate the symptom of RA by reducing the Th17 cells, and increasing the number of CD4^+^CD25^+^ Treg cells at the site of inflammation. As a matter of fact, tocilizumab corrected the balance of Th17/Treg cells in RA patients (38).

IL-1 is a central pro-inflammatory cytokine that impacts on a number of cell types and subsequently induces bone and cartilage destruction. IL-1 is another important biological that modulates RA pathology. Human recombinant IL-1 receptor antagonist (IL-1ra) can block the IL-1 mediated effects, and restore the balance of Th17/Treg cells. Even though the exact mechanisms remain largely unknown, the use of anakinra, an IL-1ra, as a therapy in RA was effective and safe (39). Treg cells highly express CTLA-4, which controls the suppressive function of Treg cells (40). CTLA-4 is also expressed on activated T cells, however, binding to its ligands induces opposite effects in effector and Treg cells. For example, ligation of CTLA-4 with CD80 (B7-1) and CD86 (B7-2) inhibits the function of Teffs but augments the suppressive capacity of Treg cells. CTLA-4-Ig (also known as abatacept) is a human fusion protein that consists of the extracellular domain of CTLA-4 and the Fc portion of IgG1, which is used for the treatments of RA patients.

**Figure 1:**
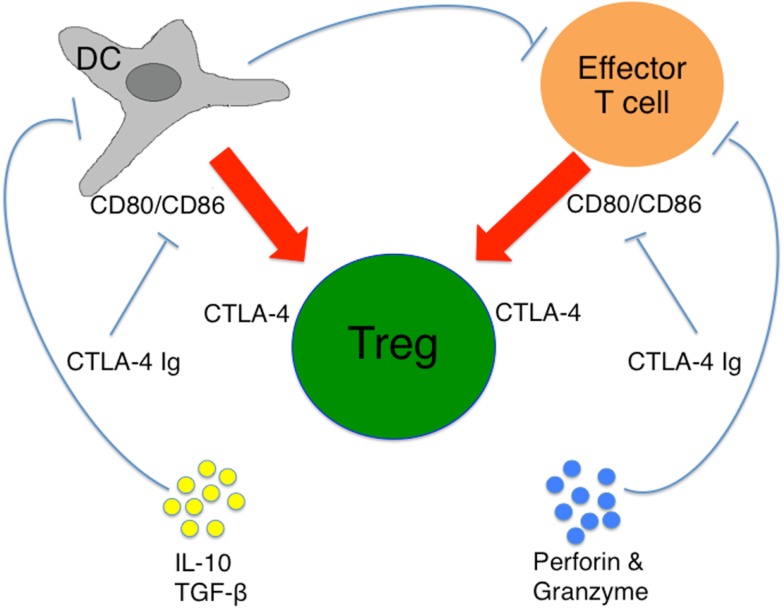
**Suppressive mechanisms of Treg cells**. Treg cells mediate their suppressive activity by direct cell–cell contact mediated by CTLA-4 on both Teffs and APCs, e.g., DCs. Suppressive cytokines (IL-10 and TGF-β) suppresses DC maturation and function. Treg cells also destroy Teffs through secreting perforin and granzyme-A.

In addition, nTregs and iTreg cells can directly kill autologous target cells through a CD18 and perforin-dependent manner (41–43). Upon activation, nTreg cells predominantly express granzyme-A while iTreg cells express granzyme B. Both subtypes of Treg cells exhibit perforin-dependent cytotoxicity against a variety of autologous target cells, including CD4^+^ and CD8^+^ T cells, CD14^+^ monocytes, and DCs. The mechanism by which the Treg cell subsets recognize their targets is unclear; however, several lines of evidence suggest that it is an MHC/TCR independent process. The potential suppressive mechanisms of Treg cells are briefly described in Figure 1.

## Treg Cell-Based Therapies in RA

Regulatory T cells play a central role in controlling autoimmunity. Deficiencies in the functions of Treg cells have been identified in a number of autoimmune diseases including RA. A number of studies suggested that stem cell therapy or biological therapy induced a potent population of CD4^+^CD25^+^ Treg cells in patients with RA (44); however, the deficit of nTreg cells persisted in the patients even after the treatment with anti-TNF-α (45). CD4^+^CD25^+^ Treg cells isolated from active RA patients were capable of suppressing the proliferation of conventional T cells; however, these Treg cells could not suppress the cytokine production. It is suggested that reduced expression and functional abnormalities in Treg cells associated with CTLA-4 might account for the defect of Treg cells in patients with RA (25).

After the success of the biological therapies in RA, new therapeutic adventures with the goal to ultimately reach complete clinical remission become more attractive. For reestablish the tolerance by utilizing Treg cells, numerous studies have been performed on Treg cell-based therapies. This approach can be reached by expansion of CD4^+^CD25^+^ Treg cells *ex vivo* or induction of Treg cells *in vivo*. Because of the inherent resistance of Treg cells to exogenous expansion, and large numbers of Treg cells essentially required to perform the cell-based therapies, it is critical to isolate a high number of Treg cells for exogenous expansion, and the following adoptive transfer. However, caution is needed when translating *in vitro* induction of Treg cells to *in vivo* application. To make sure that Treg cells suppress Teffs at the site of inflammation, a number of strategies have been suggested to regulate the numbers and functionality of Treg cells such as ectopic expression or the acetylation modulation of Foxp3 (46).

Embryonic stem cells (ESCs) or induced pluripotent stem cells (iPSCs) have the strong potential to differentiate into a number of different cell types in the body. Different types of blood cells including myeloid and lymphoid lineages can arise from ESCs or iPSCs. The approach to obtain ESCs from patients is not feasible. HSCs have a higher potential to pass the bone marrow barrier and travel in the blood, which allows HSCs to be easily and safely harvested from the patient blood following mobilization with hematopoietic growth factors. Thus, the use of HSC for therapeutic purposes has been widely applied in clinic, especially in HSC transplantations. In addition, HSCs also can be generated from differentiated cultures from ESCs and fetal-derived embryonic germ stem cells (47). However, compared to pluripotent ESCs or iPSCs, HSCs or MSCs have less potential for self-renewal, and the differentiation declines in response to differentiation signals with each cycle (48). In fact, the ability to expand numbers *in vivo* or *in vitro* would be a huge boost to all current and future medical uses of HSCs and MSCs. In contrast, iPSCs can be easily generated from patients’ somatic cells by transduction of various transcription factors and exhibit characteristics identical to those of ESCs (49, 50). Many genetic methods as well as the protein-based approach have been developed to produce iPSC with potentially reduced risks, including that of immunogenicity and tumorigenicity (51–53). This approach provides an opportunity to generate patient- or disease-specific iPSCs (54–56). Because of the plasticity and the potential for an unlimited capacity for self-renewal, iPSCs have high potential for advancing the field of cell-based therapies (57, 58). Recent results have shown that programing of functional CD4^+^ Treg cells or CD8^+^ cytotoxic T lymphocytes from iPSCs can be used for adoptive immunotherapy of cancers and autoimmune arthritis (59–62).

Embryonic stem cells and iPSCs are exposed to a number of signals responsible for their progression. Although the exact signals are not fully understood, part of the mechanism known to be critical for directing T-cell fate occurs via the Notch signaling pathway (63, 64). Notch is evolutionarily conserved regulating cell fate decisions in a number of cell and tissue types. Ligand binding by members of the Jagged or Delta-like families results in the proteolytic cleavage and release of the intracellular fragment of the Notch heterodimer. Translocation to the nucleus then allows for its regulation of gene expression. Notch-1, specifically, has been shown to be critical for the establishment of T-cell fate. Loss of function results in the blockade of T cell development and enhanced B cell production, while over-expression results in the blockade of B cell lymphopoiesis and leads to the generation of T cells (64). However, the intracellular signaling pathways by which Notch signaling regulates Ag-specific PSC–Treg differentiation remain unknown. Genetic modification of iPSCs with the Foxp3 gene and an antigen-specific TCR as well as stimulation with an *in vitro* Notch ligand may direct iPSC differentiation into antigen-specific Treg cells, which produce suppressive cytokines and inhibit other immune cell activities. Adoptive transfer of iPSC-derived Treg cells has been shown to suppress the development of arthritis in a murine model (62).

## Immunosuppression by Treg Cell Therapy

The transfer of *ex vivo* expanded Treg cells into the patients is presently the focus of intense research to treat autoimmune disorders and to suppress the occurrence of graft-versus-host disease (GVHD) after bone marrow transplantation (65, 66). *Ex vivo* expanded Treg cells were dramatically useful in the prevention of autoimmune diseases, and in many cases, in the treatments of ongoing inflammatory and autoimmune disorders in mice (4). A number of mechanisms have been suggested for Treg cell-mediated suppression, including secretion of immunosuppressive cytokines from the Treg cells, cell contact dependent suppression, and functional modification or killing of APCs. However, it is not clear whether Treg cells *in vivo* use same or different mechanisms. Nevertheless, a number of key issues need to be considered prior to their clinical use. First, because of the heterogeneity of Foxp3^+^ Treg cells, it is important to determine the optimal Treg cells that should be isolated for expansion. Expansion of Foxp3-expressing T cells is based on isolation of CD25 and CD127; this method usually results in contamination of non-regulatory CD45RO^+^Foxp3^low^ T cells. CD45RO^+^FoxP3^low^ cells are capable of producing pro-inflammatory cytokines, and likely constitute 30–50% of the Foxp3^+^CD4^+^ T cells (67). Because FoxP3 expression is crucial for the suppressive activity of Treg cells, and CD45RA^+^Foxp3^low^ naive Treg cells have the highest capacity of maintaining the expression of Foxp3 after expansion, the subset of CD45RA^+^Foxp3^low^ naive Treg cells is the best population of choice for isolation (68).

Second, the apoptotic properties of Treg cells should be investigated because these cells may be used to modulate the ratio of Treg to Teff cells. Treg cells are prone to die by apoptosis (69) and it is hard to expand in cultures with high dose of IL-2 (67). In addition, naive Treg cells are easier to expand and develop a high frequency of functional Foxp3^+^ Treg cells; the use of CD45RA^+^Foxp3^low^ naive Treg cells is the best choice for the expansion of Treg cells. It has been shown that purified naive Treg cells, when cultured with high-dose IL-2, could give rise to inflammatory cytokine producing T cells (70). Therefore, certain cytokines and chemicals need to be determined to enable the expansion of functional Treg cells, which will not secrete pro-inflammatory cytokines. Rapamycin (also known as Sirolimus/Rapamune) likely be useful as a component as it substantially increases the purity of Treg cells by eliminating non-Treg cells (71).

To onset the development and pathogenesis of arthritis in mice, a number of cytokines are regarded to play a crucial role (72). Among them, IL-17 has an important role; IL-17 is a T cell derived pro-inflammatory cytokine produced by TCRα/β^+^CD4^−^CD8^−^ thymocytes, as well as activated CD4^+^ and CD4^+^CD45RO^+^ memory T cells (73). IL-17 is involve in the development of RA because of the existence in the synovial fluid of RA patients and the production from T cell clones established from RA patients (74). IL-17 played a role in the early and late stages of collagen induced arthritis (75). Adoptively transfer of engineered Treg cells substantially decreased inflammatory knee swelling and a significant reduction of Th17 cells in the draining lymph nodes (76). Treg cells were capable of limiting the detrimental effects of Th17 cells by reducing accumulation at the site of inflammation and in the draining lymph nodes (77). However, Treg cells might be inefficient suppressors of Th17 cells (25, 26). Taken together, a successful therapy using Foxp3^+^ Treg cells (both nTreg and iTreg cells) depends on how Treg cells are prepared, the purity of the Treg cells, and how the Treg cells are maintained as a functionally stable cells *in vivo* as well as *in vitro* expanded.

For a successful and potential immunotherapy using Treg cells, Ag specificity of Treg cells are highly recommended. The importance of antigen specificity is a strong indication where high doses of polyclonal Treg cells fail to reverse ongoing autoimmunity. Antigen specificity is required for Treg cell to home or retained at the appropriate site and exerts active suppression. A retroviral transduction protocol in which OTII TCR genes were transduced in primary Treg cells and in CD4^+^ T cells allows these cells to redirect the specificity without the needs for lengthy *in vitro* culture. Within 3 days, their redirected Treg cells and converted CD4^+^ T cells were available for adoptive transfer into recipient mice, where they engrafted efficiently, maintained their phenotype, and suppressed arthritic joint inflammation in an antigen dependent fashion (76). Foxp3 transduction of naive CD4^+^ T cells can convert these cells into functional Foxp3^+^ Treg cells that prevent or suppress the development of a number of autoimmune diseases in animals (78–80). Because of the excellent capacity of stem cell-derived Treg cells in suppression of autoimmunity (62), generation of antigen-specific Foxp3^+^ Treg cells from stem cells such as iPSCs and HSCs may open a new area in Treg cell-based immunotherapy in transplantation and autoimmune disorders.

## Advantages and Disadvantages for the Use of Treg Cell-Based Therapy

Reestablishing self-tolerance in autoimmunity depends on self-reactive Treg cells. The advantages of Treg cell-based immunotherapy over conventional treatments are numerous. These benefits include: (1) the potential for antigen specificity with the lack of general immunosuppression; (2) the possibility of inducing long-lasting regulation *in vivo*; and (3) custom-made product that can be designed for each patient, with very limited or absent side effects. However, due to the lack of Treg cell specific surface antigens, purification of a more comprehensive Treg cell subset increases the contamination by the non-regulatory Teffs. Although the therapeutic potential for Treg cell-based immunotherapy is now well established in animal models (81–87), to date, such therapy has not been directly applied to suppress autoimmunity in humans.

There are a number of problems and limitations in generating a large number of Treg cells, in spite of the growing number of methods for isolating Treg cells. No approach or method has been established to isolate the population of Treg cells with 100% specificity. In addition to the issue of isolation, the survival of Treg cells is another critical factor in Treg cell-based therapies. Treg cells are disposed to apoptosis in the absence of certain cytokines (e.g., IL-2) since Treg cells do not secrete IL-2, and are essentially dependent on other cells (88). Also, Treg cells are prone to apoptosis because of the low expression of the bcl-2 family proteins. Bcl-xL is an anti-apoptotic protein that can sustain the survival of T lymphocytes (88). β-catenin promoted the survival of Treg cells *in vitro* without changing their anergic state or suppressive function by regulating expression of anti-apoptotic bcl-xL (89). Indeed, bcl-xL is involved in the development and function of Treg cells by inducing the expression of Foxp3, CTLA-4, TGF-β, and repressing the programed death receptor-1 (PD-I) expression (90, 91). Foxp3 and bcl-xL cooperatively promote the survival of Treg cells and prevent arthritis development because of the increased survival of Treg cells (80). These approaches likely induce the differentiation and survival of Treg cells, and result in the persistence of Treg cells.

In addition, there are several unsettled issues regarding the function of Treg cells in rheumatic diseases. These unresolved questions significantly limit the potential use of Treg cell-based therapies in clinic. For instance, it remains unclear whether the existed effects of Treg cell-based therapies redirect the fundamental mechanism that donates to medical progress. In fact, the differences in the compartment of Treg cells may not be directly involved in the improvement of symptoms. Assessment of the function of Treg cells is also an obstacle, because there is no specific surface marker of Treg cells in humans, and highly activated T cells also transiently express Foxp3. Furthermore, the accessibility of the site of inflammation in humans is limited. Of note, most investigations are studies in the periphery, which possibly will not uncover the interactions occurring at the site of inflammation (92).

## Conclusion

Due to the plasticity and possibly infinite ability for self-renewal, stem cell-derived CD4^+^CD25^+^ Treg cells likely are applicable for Treg cell-based immunotherapy, such as autoimmune disorders. Several questions remain: (i) how to direct the differentiation of Treg cells, (ii) how to obtain a large number of functional Treg cells, and (iii) which type of stem cells can be feasibly applicable for individual use. Stem cell or their offspring likely is an optimal option for the use of Treg cell-based therapies. iPSCs have great potential as a source for the generation of antigen-specific Treg cells to be utilized in personalized therapies. However, significant challenges remain to bring the use of stem cell-based technologies into the clinic to treat destructive chronic diseases like RA.

More also needs to be known about the effects of standard therapy on Treg cells, because the precise contribution of Treg cells to the pathogenesis of RA remains unclear, even Treg cells play an important role. Regarding MTX, it is still controversial that Treg cells are impacted by the treatments of MTX. With regards to targeting the pro-inflammatory cytokines TNF-α, IL-6, IL-1, and the costimulatory molecule CTLA-4, most investigations strengthen a stimulating effect of blocking activity on Treg cells. This underscores the importance of further studies on the effects of current therapies on the function of Treg cells, which could eventually lead to the therapeutic applications by utilizing Treg cells.

## Conflict of Interest Statement

The authors declare that the research was conducted in the absence of any commercial or financial relationships that could be construed as a potential conflict of interest.

## References

[1] Brennan FM McInnes IB Evidence that cytokines play a role in rheumatoid arthritis. J Clin Invest (2008) 118(11):3537–45 10.1172/JCI36389 18982160 PMC2575731

[2] Sakaguchi S Naturally arising Foxp3-expressing CD25+CD4+ regulatory T cells in immunological tolerance to self and non-self. Nat Immunol (2005) 6(4):345–52 10.1038/ni1178 15785760

[3] Van VQ Darwiche J Raymond M Lesage S Bouguermouh S Rubio M Cutting edge: CD47 controls the in vivo proliferation and homeostasis of peripheral CD4+ CD25+ Foxp3+ regulatory T cells that express CD103. J Immunol (2008) 181(8):5204–8 10.4049/jimmunol.181.8.5204 18832672

[4] Sakaguchi S Yamaguchi T Nomura T Ono M Regulatory T cells and immune tolerance. Cell (2008) 133(5):775–87 10.1016/j.cell.2008.05.009 18510923

[5] Bollyky PL Wu RP Falk BA Lord JD Long SA Preisinger A ECM components guide IL-10 producing regulatory T-cell (TR1) induction from effector memory T-cell precursors. Proc Natl Acad Sci U S A (2011) 108(19):7938–43 10.1073/pnas.1017360108 21518860 PMC3093524

[6] Li R Perez N Karumuthil-Melethil S Prabhakar BS Holterman MJ Vasu C Enhanced engagement of CTLA-4 induces antigen-specific CD4+CD25+Foxp3+ and CD4+CD25- TGF-beta 1+ adaptive regulatory T cells. J Immunol (2007) 179(8):5191–203 10.4049/jimmunol.179.8.5191 17911604

[7] Chaudhry A Rudra D Treuting P Samstein RM Liang Y Kas A CD4+ regulatory T cells control TH17 responses in a Stat3-dependent manner. Science (2009) 326(5955):986–91 10.1126/science.1172702 19797626 PMC4408196

[8] Riley JL June CH Blazar BR Human T regulatory cell therapy: take a billion or so and call me in the morning. Immunity (2009) 30(5):656–65 10.1016/j.immuni.2009.04.006 19464988 PMC2742482

[9] Wan YY Flavell RA Regulatory T-cell functions are subverted and converted owing to attenuated Foxp3 expression. Nature (2007) 445(7129):766–70 10.1038/nature05479 17220876

[10] Williams LM Rudensky AY Maintenance of the Foxp3-dependent developmental program in mature regulatory T cells requires continued expression of Foxp3. Nat Immunol (2007) 8(3):277–84 10.1038/ni1437 17220892

[11] Kim HP Leonard WJ CREB/ATF-dependent T cell receptor-induced FoxP3 gene expression: a role for DNA methylation. J Exp Med (2007) 204(7):1543–51 10.1084/jem.20070109 17591856 PMC2118651

[12] Ouyang W Beckett O Ma Q Paik JH DePinho RA Li MO Foxo proteins cooperatively control the differentiation of Foxp3+ regulatory T cells. Nat Immunol (2010) 11(7):618–27 10.1038/ni.1884 20467422

[13] Yao Z Kanno Y Kerenyi M Stephens G Durant L Watford WT Nonredundant roles for Stat5a/b in directly regulating Foxp3. Blood (2007) 109(10):4368–75 10.1182/blood-2006-11-055756 17227828 PMC1885496

[14] Gavin MA Torgerson TR Houston E DeRoos P Ho WY Stray-Pedersen A Single-cell analysis of normal and FOXP3-mutant human T cells: FOXP3 expression without regulatory T cell development. Proc Natl Acad Sci U S A (2006) 103(17):6659–64 10.1073/pnas.0509484103 16617117 PMC1458937

[15] Duarte JH Zelenay S Bergman ML Martins AC Demengeot J Natural Treg cells spontaneously differentiate into pathogenic helper cells in lymphopenic conditions. Eur J Immunol (2009) 39(4):948–55 10.1002/eji.200839196 19291701

[16] Komatsu N Mariotti-Ferrandiz ME Wang Y Malissen B Waldmann H Hori S Heterogeneity of natural Foxp3+ T cells: a committed regulatory T-cell lineage and an uncommitted minor population retaining plasticity. Proc Natl Acad Sci U S A (2009) 106(6):1903–8 10.1073/pnas.0811556106 19174509 PMC2644136

[17] Yang XO Nurieva R Martinez GJ Kang HS Chung Y Pappu BP Molecular antagonism and plasticity of regulatory and inflammatory T cell programs. Immunity (2008) 29(1):44–56 10.1016/j.immuni.2008.05.007 18585065 PMC2630532

[18] Oldenhove G Bouladoux N Wohlfert EA Hall JA Chou D Dos Santos L Decrease of Foxp3+ Treg cell number and acquisition of effector cell phenotype during lethal infection. Immunity (2009) 31(5):772–86 10.1016/j.immuni.2009.10.001 19896394 PMC2814877

[19] Dang EV Barbi J Yang HY Jinasena D Yu H Zheng Y Control of T(H)17/T(reg) balance by hypoxia-inducible factor 1. Cell (2011) 146(5):772–84 10.1016/j.cell.2011.07.033 21871655 PMC3387678

[20] van der Horst A de Vries-Smits AM Brenkman AB van Triest MH van den Broek N Colland F FOXO4 transcriptional activity is regulated by monoubiquitination and USP7/HAUSP. Nat Cell Biol (2006) 8(10):1064–73 10.1038/ncb1469 16964248

[21] van Loosdregt J Vercoulen Y Guichelaar T Gent YY Beekman JM van Beekum O Regulation of Treg functionality by acetylation-mediated Foxp3 protein stabilization. Blood (2010) 115(5):965–74 10.1182/blood-2009-02-207118 19996091

[22] van Loosdregt J Fleskens V Fu J Brenkman AB Bekker CP Pals CE Stabilization of the transcription factor Foxp3 by the deubiquitinase USP7 increases Treg-cell-suppressive capacity. Immunity (2013) 39(2):259–71 10.1016/j.immuni.2013.05.018 23973222 PMC4133784

[23] Mottonen M Heikkinen J Mustonen L Isomaki P Luukkainen R Lassila O CD4+ CD25+ T cells with the phenotypic and functional characteristics of regulatory T cells are enriched in the synovial fluid of patients with rheumatoid arthritis. Clin Exp Immunol (2005) 140(2):360–7 10.1111/j.1365-2249.2005.02754.x 15807863 PMC1809357

[24] Valencia X Stephens G Goldbach-Mansky R Wilson M Shevach EM Lipsky PE TNF downmodulates the function of human CD4+CD25hi T-regulatory cells. Blood (2006) 108(1):253–61 10.1182/blood-2005-11-4567 16537805 PMC1895836

[25] Flores-Borja F Jury EC Mauri C Ehrenstein MR Defects in CTLA-4 are associated with abnormal regulatory T cell function in rheumatoid arthritis. Proc Natl Acad Sci U S A (2008) 105(49):19396–401 10.1073/pnas.0806855105 19036923 PMC2614772

[26] Veldhoen M Hocking RJ Atkins CJ Locksley RM Stockinger B TGFbeta in the context of an inflammatory cytokine milieu supports de novo differentiation of IL-17-producing T cells. Immunity (2006) 24(2):179–89 10.1016/j.immuni.2006.01.001 16473830

[27] Nie H Zheng Y Li R Guo TB He D Fang L Phosphorylation of FOXP3 controls regulatory T cell function and is inhibited by TNF-alpha in rheumatoid arthritis. Nat Med (2013) 19(3):322–8 10.1038/nm.3085 23396208

[28] Coury FF Weinblatt ME Clinical trials to establish methotrexate as a therapy for rheumatoid arthritis. Clin Exp Rheumatol (2010) 28(5 Suppl 61):S9–12 21044426

[29] Boyce EG Halilovic J Stan-Ugbene O Golimumab: review of the efficacy and tolerability of a recently approved tumor necrosis factor-alpha inhibitor. Clin Ther (2010) 32(10):1681–703 10.1016/j.clinthera.2010.09.003 21194591

[30] De Stefano R Frati E Nargi F Baldi C Menza L Hammoud M Comparison of combination therapies in the treatment of rheumatoid arthritis: leflunomide-anti-TNF-alpha versus methotrexate-anti-TNF-alpha. Clin Rheumatol (2010) 29(5):517–24 10.1007/s10067-009-1349-y 20082236

[31] Mori S Sugimoto M Is continuation of anti-tumor necrosis factor-alpha therapy a safe option for patients who have developed pulmonary mycobacterial infection? Case presentation and literature review. Clin Rheumatol (2012) 31(2):203–10 10.1007/s10067-011-1902-3 22170032

[32] van Vollenhoven RF Geborek P Forslind K Albertsson K Ernestam S Petersson IF Conventional combination treatment versus biological treatment in methotrexate-refractory early rheumatoid arthritis: 2 year follow-up of the randomised, non-blinded, parallel-group Swefot trial. Lancet (2012) 379(9827):1712–20 10.1016/S0140-6736(12)60027-0 22464340

[33] Soliman MM Hyrich KL Lunt M Watson KD Symmons DP Ashcroft DM Effectiveness of rituximab in patients with rheumatoid arthritis: observational study from the British Society for Rheumatology Biologics Register. J Rheumatol (2012) 39(2):240–6 10.3899/jrheum.110610 22174201

[34] Yang L Anderson DE Baecher-Allan C Hastings WD Bettelli E Oukka M IL-21 and TGF-beta are required for differentiation of human T(H)17 cells. Nature (2008) 454(7202):350–2 10.1038/nature07021 18469800 PMC2760130

[35] Miossec P Interleukin-17 in fashion, at last: ten years after its description, its cellular source has been identified. Arthritis Rheum (2007) 56(7):2111–5 10.1002/art.22733 17599728

[36] Koenders MI Lubberts E Oppers-Walgreen B van den Bersselaar L Helsen MM Di Padova FE Blocking of interleukin-17 during reactivation of experimental arthritis prevents joint inflammation and bone erosion by decreasing RANKL and interleukin-1. Am J Pathol (2005) 167(1):141–9 10.1016/S0002-9440(10)62961-6 15972960 PMC1603454

[37] Navarro-Millan I Singh JA Curtis JR Systematic review of tocilizumab for rheumatoid arthritis: a new biologic agent targeting the interleukin-6 receptor. Clin Ther (2012) 34(4):788–802e3 10.1016/j.clinthera.2012.02.014 22444783 PMC3805022

[38] Samson M Audia S Janikashvili N Ciudad M Trad M Fraszczak J Brief report: inhibition of interleukin-6 function corrects Th17/Treg cell imbalance in patients with rheumatoid arthritis. Arthritis Rheum (2012) 64(8):2499–503 10.1002/art.34477 22488116

[39] Quartier P Allantaz F Cimaz R Pillet P Messiaen C Bardin C A multicentre, randomised, double-blind, placebo-controlled trial with the interleukin-1 receptor antagonist anakinra in patients with systemic-onset juvenile idiopathic arthritis (ANAJIS trial). Ann Rheum Dis (2011) 70(5):747–54 10.1136/ard.2010.134254 21173013 PMC3070271

[40] Wing K Onishi Y Prieto-Martin P Yamaguchi T Miyara M Fehervari Z CTLA-4 control over Foxp3+ regulatory T cell function. Science (2008) 322(5899):271–5 10.1126/science.1160062 18845758

[41] Grossman WJ Verbsky JW Barchet W Colonna M Atkinson JP Ley TJ Human T regulatory cells can use the perforin pathway to cause autologous target cell death. Immunity (2004) 21(4):589–601 10.1016/j.immuni.2004.09.002 15485635

[42] Singh K Gatzka M Peters T Borkner L Hainzl A Wang H Reduced CD18 levels drive regulatory T cell conversion into Th17 cells in the CD18hypo PL/J mouse model of psoriasis. J Immunol (2013) 190(6):2544–53 10.4049/jimmunol.1202399 23418628

[43] Marski M Kandula S Turner JR Abraham C CD18 is required for optimal development and function of CD4+CD25+ T regulatory cells. J Immunol (2005) 175(12):7889–97 10.4049/jimmunol.175.12.7889 16339524

[44] Gonzalez-Rey E Gonzalez MA Varela N O’Valle F Hernandez-Cortes P Rico L Human adipose-derived mesenchymal stem cells reduce inflammatory and T cell responses and induce regulatory T cells in vitro in rheumatoid arthritis. Ann Rheum Dis (2010) 69(1):241–8 10.1136/ard.2008.101881 19124525

[45] Nadkarni S Mauri C Ehrenstein MR Anti-TNF-alpha therapy induces a distinct regulatory T cell population in patients with rheumatoid arthritis via TGF-beta. J Exp Med (2007) 204(1):33–9 10.1084/jem.20061531 17200409 PMC2118431

[46] Kornete M Piccirillo CA Functional crosstalk between dendritic cells and Foxp3(+) regulatory T cells in the maintenance of immune tolerance. Front Immunol (2012) 3:165 10.3389/fimmu.2012.00165 22737152 PMC3381230

[47] Ledran MH Krassowska A Armstrong L Dimmick I Renstrom J Lang R Efficient hematopoietic differentiation of human embryonic stem cells on stromal cells derived from hematopoietic niches. Cell Stem Cell (2008) 3(1):85–98 10.1016/j.stem.2008.06.001 18593561

[48] Himburg HA Muramoto GG Daher P Meadows SK Russell JL Doan P Pleiotrophin regulates the expansion and regeneration of hematopoietic stem cells. Nat Med (2010) 16(4):475–82 10.1038/nm.2119 20305662 PMC3689427

[49] Nakagawa M Koyanagi M Tanabe K Takahashi K Ichisaka T Aoi T Generation of induced pluripotent stem cells without Myc from mouse and human fibroblasts. Nat Biotechnol (2008) 26(1):101–6 10.1038/nbt1374 18059259

[50] Kim JB Sebastiano V Wu G Arauzo-Bravo MJ Sasse P Gentile L Oct4-induced pluripotency in adult neural stem cells. Cell (2009) 136(3):411–9 10.1016/j.cell.2009.01.023 19203577

[51] Li Z Yang CS Nakashima K Rana TM Small RNA-mediated regulation of iPS cell generation. EMBO J (2011) 30(5):823–34 10.1038/emboj.2011.2 21285944 PMC3049216

[52] Zhao T Zhang ZN Rong Z Xu Y Immunogenicity of induced pluripotent stem cells. Nature (2011) 474(7350):212–5 10.1038/nature10135 21572395

[53] Azevedo JL Feldman RA Tinkering with transcription factors uncovers plasticity of somatic cells. Genes Cancer (2010) 1(11):1089–99 10.1177/1947601911401908 21779433 PMC3092276

[54] Soldner F Hockemeyer D Beard C Gao Q Bell GW Cook EG Parkinson’s disease patient-derived induced pluripotent stem cells free of viral reprogramming factors. Cell (2009) 136(5):964–77 10.1016/j.cell.2009.02.013 19269371 PMC2787236

[55] Raya A Rodriguez-Piza I Guenechea G Vassena R Navarro S Barrero MJ Disease-corrected haematopoietic progenitors from Fanconi anaemia induced pluripotent stem cells. Nature (2009) 460(7251):53–9 10.1038/nature08129 19483674 PMC2720823

[56] Ebert AD Yu J Rose FF Jr Mattis VB Lorson CL Thomson JA Induced pluripotent stem cells from a spinal muscular atrophy patient. Nature (2009) 457(7227):277–80 10.1038/nature07677 19098894 PMC2659408

[57] Robinton DA Daley GQ The promise of induced pluripotent stem cells in research and therapy. Nature (2012) 481(7381):295–305 10.1038/nature10761 22258608 PMC3652331

[58] Sandhaus RA Gene therapy meets stem cells. N Engl J Med (2012) 366(6):567–9 10.1056/NEJMcibr1113400 22316453

[59] Lei F Haque R Weiler L Vrana KE Song J T lineage differentiation from induced pluripotent stem cells. Cell Immunol (2009) 260(1):1–5 10.1016/j.cellimm.2009.09.005 19811778

[60] Lei F Zhao B Haque R Xiong X Budgeon L Christensen ND In vivo programming of tumor antigen-specific T lymphocytes from pluripotent stem cells to promote cancer immunosurveillance. Cancer Res (2011) 71(14):4742–7 10.1158/0008-5472.CAN-11-0359 21628492

[61] Lei F Haque R Xiong X Song J Directed differentiation of induced pluripotent stem cells towards T lymphocytes. J Vis Exp (2012) (63):e3986 10.3791/3986 22617911 PMC3389997

[62] Haque R Lei F Xiong X Bian Y Zhao B Wu Y Programming of regulatory T cells from pluripotent stem cells and prevention of autoimmunity. J Immunol (2012) 189(3):1228–36 10.4049/jimmunol.1200633 22732595 PMC3401327

[63] Schmitt TM de Pooter RF Gronski MA Cho SK Ohashi PS Zuniga-Pflucker JC Induction of T cell development and establishment of T cell competence from embryonic stem cells differentiated in vitro. Nat Immunol (2004) 5(4):410–7 10.1038/ni1055 15034575

[64] Dervovic DD Liang HC Cannons JL Elford AR Mohtashami M Ohashi PS Cellular and molecular requirements for the selection of in vitro-generated CD8 T cells reveal a role for notch. J Immunol (2013) 191(4):1704–15 10.4049/jimmunol.1300417 23851691 PMC3801448

[65] Miyara M Wing K Sakaguchi S Therapeutic approaches to allergy and autoimmunity based on FoxP3+ regulatory T-cell activation and expansion. J Allergy Clin Immunol (2009) 123(4):749–55;quiz56–7. 10.1016/j.jaci.2009.03.001 19348913

[66] Trzonkowski P Bieniaszewska M Juscinska J Dobyszuk A Krzystyniak A Marek N First-in-man clinical results of the treatment of patients with graft versus host disease with human ex vivo expanded CD4+CD25+CD127- T regulatory cells. Clin Immunol (2009) 133(1):22–6 10.1016/j.clim.2009.06.001 19559653

[67] Putnam AL Brusko TM Lee MR Liu W Szot GL Ghosh T Expansion of human regulatory T-cells from patients with type 1 diabetes. Diabetes (2009) 58(3):652–62 10.2337/db08-1168 19074986 PMC2646064

[68] Hoffmann P Eder R Kunz-Schughart LA Andreesen R Edinger M Large-scale in vitro expansion of polyclonal human CD4(+)CD25high regulatory T cells. Blood (2004) 104(3):895–903 10.1182/blood-2004-01-0086 15090447

[69] Fritzsching B Oberle N Eberhardt N Quick S Haas J Wildemann B In contrast to effector T cells, CD4+CD25+FoxP3+ regulatory T cells are highly susceptible to CD95 ligand- but not to TCR-mediated cell death. J Immunol (2005) 175(1):32–6 10.4049/jimmunol.175.1.32 15972628

[70] Hoffmann P Eder R Boeld TJ Doser K Piseshka B Andreesen R Only the CD45RA+ subpopulation of CD4+CD25high T cells gives rise to homogeneous regulatory T-cell lines upon in vitro expansion. Blood (2006) 108(13):4260–7 10.1182/blood-2006-06-027409 16917003

[71] Battaglia M Stabilini A Migliavacca B Horejs-Hoeck J Kaupper T Roncarolo MG Rapamycin promotes expansion of functional CD4+CD25+FOXP3+ regulatory T cells of both healthy subjects and type 1 diabetic patients. J Immunol (2006) 177(12):8338–47 10.4049/jimmunol.177.12.8338 17142730

[72] Campbell IK O’Donnell K Lawlor KE Wicks IP Severe inflammatory arthritis and lymphadenopathy in the absence of TNF. J Clin Invest (2001) 107(12):1519–27 10.1172/JCI12724 11413159 PMC200197

[73] Aggarwal S Gurney AL IL-17: prototype member of an emerging cytokine family. J Leukoc Biol (2002) 71(1):1–8 10.1189/jlb.1938-3673 11781375

[74] Aarvak T Chabaud M Miossec P Natvig JB IL-17 is produced by some proinflammatory Th1/Th0 cells but not by Th2 cells. J Immunol (1999) 162(3):1246–51 9973376

[75] Lubberts E Joosten LA Oppers B van den Bersselaar L Coenen-de Roo CJ Kolls JK IL-1-independent role of IL-17 in synovial inflammation and joint destruction during collagen-induced arthritis. J Immunol (2001) 167(2):1004–13 10.4049/jimmunol.167.2.1004 11441109

[76] Wright GP Notley CA Xue SA Bendle GM Holler A Schumacher TN Adoptive therapy with redirected primary regulatory T cells results in antigen-specific suppression of arthritis. Proc Natl Acad Sci U S A (2009) 106(45):19078–83 10.1073/pnas.0907396106 19884493 PMC2776462

[77] Lohr J Knoechel B Wang JJ Villarino AV Abbas AK Role of IL-17 and regulatory T lymphocytes in a systemic autoimmune disease. J Exp Med (2006) 203(13):2785–91 10.1084/jem.20061341 17130300 PMC2118184

[78] Jaeckel E von Boehmer H Manns MP Antigen-specific FoxP3-transduced T-cells can control established type 1 diabetes. Diabetes (2005) 54(2):306–10 10.2337/diabetes.54.2.306 15591438

[79] Qian Z Latham KA Whittington KB Miller DC Brand DD Rosloniec EF Engineered regulatory T cells coexpressing MHC class II:peptide complexes are efficient inhibitors of autoimmune T cell function and prevent the development of autoimmune arthritis. J Immunol (2013) 190(11):5382–91 10.4049/jimmunol.1300024 23630354 PMC3673549

[80] Haque R Lei F Xiong X Wu Y Song J FoxP3 and Bcl-xL cooperatively promote regulatory T cell persistence and prevention of arthritis development. Arthritis Res Ther (2010) 12(2):R66 10.1186/ar2983 20384988 PMC2888221

[81] Ruzek MC Waire JS Hopkins D Lacorcia G Sullivan J Roberts BL Characterization of in vitro antimurine thymocyte globulin-induced regulatory T cells that inhibit graft-versus-host disease in vivo. Blood (2008) 111(3):1726–34 10.1182/blood-2007-08-106526 18025149

[82] Samy ET Wheeler KM Roper RJ Teuscher C Tung KS Cutting edge: autoimmune disease in day 3 thymectomized mice is actively controlled by endogenous disease-specific regulatory T cells. J Immunol (2008) 180(7):4366–70 10.4049/jimmunol.180.7.4366 18354156

[83] Lages CS Suffia I Velilla PA Huang B Warshaw G Hildeman DA Functional regulatory T cells accumulate in aged hosts and promote chronic infectious disease reactivation. J Immunol (2008) 181(3):1835–48 10.4049/jimmunol.181.3.1835 18641321 PMC2587319

[84] Huter EN Stummvoll GH DiPaolo RJ Glass DD Shevach EM Cutting edge: antigen-specific TGF beta-induced regulatory T cells suppress Th17-mediated autoimmune disease. J Immunol (2008) 181(12):8209–13 10.4049/jimmunol.181.12.8209 19050237 PMC2788513

[85] Kryczek I Liu R Wang G Wu K Shu X Szeliga W FOXP3 defines regulatory T cells in human tumor and autoimmune disease. Cancer Res (2009) 69(9):3995–4000 10.1158/0008-5472.CAN-08-3804 19383912

[86] Semitekolou M Alissafi T Aggelakopoulou M Kourepini E Kariyawasam HH Kay AB Activin-A induces regulatory T cells that suppress T helper cell immune responses and protect from allergic airway disease. J Exp Med (2009) 206(8):1769–85 10.1084/jem.20082603 19620629 PMC2722168

[87] Reynolds AD Stone DK Hutter JA Benner EJ Mosley RL Gendelman HE Regulatory T cells attenuate Th17 cell-mediated nigrostriatal dopaminergic neurodegeneration in a model of Parkinson’s disease. J Immunol (2010) 184(5):2261–71 10.4049/jimmunol.0901852 20118279 PMC2824790

[88] Pandiyan P Zheng L Ishihara S Reed J Lenardo MJ CD4+CD25+Foxp3+ regulatory T cells induce cytokine deprivation-mediated apoptosis of effector CD4+ T cells. Nat Immunol (2007) 8(12):1353–62 10.1038/ni1536 17982458

[89] Ding Y Shen S Lino AC Curotto de Lafaille MA Lafaille JJ Beta-catenin stabilization extends regulatory T cell survival and induces anergy in nonregulatory T cells. Nat Med (2008) 14(2):162–9 10.1038/nm1707 18246080

[90] Sharabi A Lapter S Mozes E Bcl-xL is required for the development of functional regulatory CD4 cells in lupus-afflicted mice following treatment with a tolerogenic peptide. J Autoimmun (2010) 34(2):87–95 10.1016/j.jaut.2009.06.002 19596183

[91] Sharabi A Mozes E Bcl-xL affects the development of functional CD4 Tregs. Arthritis Res Ther (2010) 12(4):405 10.1186/ar3076 20687901 PMC2945028

[92] Wing JB Sakaguchi S Multiple Treg suppressive modules and their adaptability. Front Immunol (2012) 3:178 10.3389/fimmu.2012.00178 22754556 PMC3386489

